# Recurrent differentiated thyroid cancer: to cut or burn

**DOI:** 10.1186/1477-7819-9-89

**Published:** 2011-08-12

**Authors:** Roberto Cirocchi, Stefano Trastulli, Alessandro Sanguinetti, Lorenzo Cattorini, Piero Covarelli, Domenico Giannotti, Giorgio Di Rocco, Fabio Rondelli, Francesco Barberini, Carlo Boselli, Alberto Santoro, Nino Gullà, Adriano Redler, Nicola Avenia

**Affiliations:** 1 General and Emergency Surgical Unit. Department of Surgical Sciences, Radiology and Dentistry. University of Perugia, Perugia, Italy; 2 Endocrine Surgical Unit. Department of Surgical Sciences, Radiology and Dentistry. University of Perugia, Perugia, Italy; 3 Department of Surgical Sciences. Sapienza University of Rome, Rome, Italy

## 

The term "relapse carcinoma" is used improperly to indicate either a local or loco-regional relapse or a systematic metastatsis [1]. Local relapse (LR) after thyroidectomy for cancer is "the repetition of the neoplastic lesion in proximity of the previous intervention of excision" [2]. According to Duren [3] relapses of thyroidal carcinoma need to be classified as: local (LR): that may present itself in the residual thyroid lobe or in the thyroid bed where surgery was performed; loco-regional (RLR): that may present in the cervical lymph nodes of the central compartment or lateral-cervical nodes; and metastasis in distance (MD). The MD are frequently synchronous with LR or RLR; they have haematogenous genesis and concern most frequently the lungs and skeleton.

There is controversy over how to catergorize the relapse in the thyroidal bed with infiltrations of neighbouring organs (periodontal structures - muscles, thyroidal cartilage, cricoid, laryngeal nerves, etc. and the neighbouring organs - oesophagus, trachea, larynx). As per the classification proposed by Duren [3] these should be considered as LR, whereas according to Mozzillo and Pezzullo [1] they are categorised as RLR.

The RLR at the level of the cervical lymphnodal stations represents an ulterior problem: are these true relapses, residual cancer, or recurrence in progression? Caracò [4], in his report to the ninety-fourth Congress of the Italian Society of Surgery, specified that local recurrences are only those recurrences that are characterized by the appearance of neoplastic tissue in the thyroidal lodge, in the residual parenchyma, and in the adjacent structures, excluding the lymph nodes [5,6].

In nearly 53% of cases the relapse is reported in RLR, in 28% in LR, and in 13% the MD is present of these 6% of cases have mixed relapses [7]; the prognosis of LR is however, better than that of the others [8]. The differentiated tumors of the thyroid are slow growing and due to this rarely reach notable dimensions or result in metastasis in lymph and/or haematic systems [2]. Only 10% of patients die from differentiated thyroid cancer [9].

Most of the local relapses occur within the first five years of the excision of the primary cancer [5,6,10-12], however, the recurrence can occur as late as 20 years after the initial diagnosis and treatment [13]. An accurate evaluation of incidence of LR is possible solely with a considerable number of treated patients and lengthy follow-up that is not available at most centres and hence this kind of information can be obtained from the date from centres that have high volume of thyroid carcinoma and good follow-up like Mayo Clinic or Lahey Clinic [5,6,13] or through observational studies at several other medical centres [14].

Currently relapses represent a rare event in patients who undergo removal of thyroidal carcinoma (3-13%) [5,6,10-12,15-17].

This is due to the ever increasing frequency of total thyroidectomy for management of cancer [18]. The complete excision of the thyroidal parenchyma prevents local recurrence. Giovanni Razzaboni in "Treatise on Prognostic Surgery" (1938) stated that "The most rational operating method, so long as not free from grave consequences of another kind, remains the total extra-capsular thyroidectomy, so as is used, when possible, for the surgical removal of whatever other tumour" [19]. he further emphasized in his work published after his death in 1956 entitled "Treatise on Clinical Therapeutic Surgery" that "Only an removal of this capacity justifies, in the face of a proven malignant tumour, surgical intervention, any other incomplete or partial demolition does nothing but accelerate the ready reoccurrences, even in a very short time" [20].

The causes and modalities of onset of local relapse are multiple. There exist a series of risk factors of local relapse that correlate to the specific neoplastic illness, of these the surgical treatment used, and use of adjuvant therapies are most important.

Patients affected with thyroidal carcinoma are subdivided into risk classes on the basis of the classification systems AGES (Age, Grading, Extrathyroidal extension and tumour size) (1987), AMES (Age, distant Metastasis, Extra thyroidal extension and tumour Size) (1988) and MACIS (distant Metastasis, Age, Completeness of resection, Invasion local, tumour Size) (1993) [21]. As per these classifications low risk (mortality rate within 10 years of 1-2%)categories consists of men < 41 years and women < 51 years, with well differentiated tumor and tumors that are confined to the gland with absence of regional or distant metastasis, or older patients without extrathyroidal localization, either out of the thyroidal capsule or at distance or without extrathyroidal localization or out of the thyroidal capsule and patients with a tumor <4 cm of maximum diameter. high risk on the other hand (mortality rate within 10 years of 50-75%)are classified as all patients that do not fall into the previous category.

The most important risk factor in the onset of a local relapse is the stage of the previous tumor, particularly the local extension of the cancer and the involvement of the lymphnodes. The diameter of the neoplasia with significant risk of relapse varies from <1.5 cm (Schroder et al.: 13/50 relapses in tumors of higher diameter vs 4/55 in patients with smaller tumor) Grant et al.reported 5% of relapses within 20 years for tumours smaller then 4 cm vs 15% for larger tumours [5,6].

The spread of the cancer beyond the thyroidal capsule is another important risk factor. In Mayo Clinic study 5% of patients with intracapsular cancer relapsed within 20 years against 15% relapse in patients with extracapsular cancer. In the Lahey Clinic study relapse rates were higher at 52% (17/33) [13,22].

The presence of lymph node metastasis and follicular carcinoma (7.3% in papillary tumours vs 29.3% in follicular tumours in SICO trial) [14] are associated with an increased risk of local relapse. Moreover the age of the patient is an important variable with patietns over 45 years having higher mortality rates compared to younger patients. On the other hand, multifocality does not appear to be a significant risk factor in the development of local recurrence in patients who undergo total thyroidectomy (TT) or near total thyroidectomy (NTT) [23].

When LR appear they are associated with a poor prognosis and around 33-50% of these patients will die due to the resurgence of the illness [1]. With local relapse in the residual thyroidal tissue the outcome is less grave, compared to that involving the neighboring structures [4]. Earlier relapses have been found to have poor prognosis compared to late relapses (52.5% vs 85% [24].

In the past, at the 3-6 month the follow-up was conducted with a total body scan using a diagnostic dose of radio-iodine, TSH levels, thyroglobulin levels and antityreoglobulin antibodies. Currently, at the 3-6 month follow up is conducted with ultrasound of the neck and thyroglobulin measurements. The total body scan is no longer performed as routine as it is unable to diagnose the residual diseaseand provides no additonal information that is already provided by the levels of tyroglobulin after stimulation. The antityroglobulin antibodies estimation have false positive rate of 6% and false negative rate 1% [25].

Even at successive 6-12 month follow-up, only ultrasound and level of tyreoglbulin after stimulation with recombinant TSH is recomended. In the absence of suspected recurrence further ultrasound and biochemical check-up are conducted at 6 monthly or yearly intervals depending on the risk categories. In case of suspected local recurrence further verifications with imaging (computed tomography - CT- PET/CT, and/or total body scan) is recomended [26].

In the location of relapse tumours of the thyroid the sensitivity of TC ranges from 25 to 86% [25]. The m*agnetic resonance *imaging (MRI) is particularly useful in differentiating the neoplatic tissues from the postoperative scar tissue [25]. The sensitivity of PET in the diagnosis of thyroidal carcinoma varies from 50 to 94%; it is thus very useful in relapse cancers that do not take up I^131^. The accuracy of PET in anatomical locations is now increased with the use of PET-CT [25], which demonstrates a sensitivity of 80.7% and a specificity of 88.9% [27]. When the local recurrence or metastasis is suspected an ultrasound guided needle biopsy can be taken [1].

Currently the gold standard treatment for local relapse of thyroidal cancer is the radiometabolic treament with I^131^. The possible cures that surgery may offers in local recurrence is limited to selected cases [7]. Hence, surgical excision is advised only in cases of relapses that were not or cannot be completely treated solely with the radiometabolic treatment of I^131^.

In absence of early detection of relapse the resectibility rates are poor [28], and surgical intervention is marred by higher complecations [29,30].

The results of surgery seem to be better with local recurrences without involvement of the contiguous tissues; that constitute the minority of cases [4]. The use of intraoperative ultrasound helps in identification of the location of recurrent tumor and thus reduces the extent of the cervical dissection; this results in less postoperative complications. In patients who undergo removal of the recurrence associated with cervical dissection the prognosis is better with respect to patients in which a cervical dissection is not conducted (P = 0.0169) [31].

The results are not always disappointing; in fact Henri Redon in his monograph "Indications chirurgicales dans le traitements des cancers/Surgical Guidelines in the Treatment of Cancers" (1962) wrote: "The question of relapse. It must be re-operated and can give significant results" [32].

The ERT (external radiotherapy) is reserved for patients with inoperable relapse or tumors where I^131 ^is assumed to be ineffective [33].

Considering all the above facts and poor response of tumors to radio iodine and external therapy the multi organ resection may be considered in this select group of patients It could be a palliative resection in cases where there is a invasion of the larynx, trachea, or both organs. The infiltration of the larynx is often associated with recurring paralysis for the contemporaneous interest of a lower laryngeal nerve [34].

## Conclusions

The survival of patients with local recurrence of disease in thyroid bed is better compated to those with loco-regional or metastatic disease. Ablation of the tumor by radio-iodine appears to be a better alternative however in select cases surgical resection can be considered.

## Competing interests

The authors declare that they have no competing interests.

## Authors' contributions

CR drafted the article. TS drafted the article. SA drafted. the article. CP cooperated in writing the article and translated it into English. VN made the tables. CL searched > for the references and formatted the article. DG searched for the references and formatted the article. DRG collected patients' data. RF chose the most useful and interesting articles in literature about the field. CB searched for the references. SA searched for the references and collected the patients' consent. RA supervised the article production. NA allowed the collection of the patients' data and supervised the whole work making. All authors read and approved the final manuscript
